# Supplementary material for: Adhesions in abdomino-pelvic surgeries: A real economic impact?
Source: PLoS One. 2022 Oct 27;17(10):e0276810. doi: 10.1371/journal.pone.0276810 (PMC9612443; doi:10.1371/journal.pone.0276810)
Supplement: Attachment — Submitted filename: Courrier plos one - VF.docx [file pone.0276810.s002.docx]

Paris, 29^th^ November 2021

Dear Sir or Madam

Thank you for considering our manuscript, and for your comment.

Our manuscript is based on a retrospective study realized from a French public data. These data are obtained from PMSI database, which is the coding activities database in France for public and private hospitals. Database is managed by ATIH which is the public national body of hospital data processing. From that, available data on public website are already processed by ATIH and anonymized. Neither personal data access nor people identification are possible.

Indeed, hospitals code their activities through patient information, diagnosis and CCAM acts. Once the patient gets out of hospital, data go through a secret algorithm in order to be anonymized and become Diagnosis Related Group. From these DRG, hospitals can be paid for their activities. DRG covers the full cost of care used for patient.

Therefore, we can’t unfortunately provide any patient consent since we analyzed public data already anonymized from all public and private hospitals in France without any possibilities to be traced back to the patient.

Yours faithfully.
