# Supplementary material for: Adhesions in abdomino-pelvic surgeries: A real economic impact?
Source: PLoS One. 2022 Oct 27;17(10):e0276810. doi: 10.1371/journal.pone.0276810 (PMC9612443; doi:10.1371/journal.pone.0276810)
Supplement: Attachment — Submitted filename: Response to reviewers.docx [file pone.0276810.s003.docx]

Dear Reviewers,

We would like to thank you regarding your interest, reading time and confidence about our manuscript entitled “Adhesions in abdomino-pelvic surgeries: a real economic impact?”. We are delighted to read our work raised your attention and comments in order to increase quality of manuscript. Hereafter, you can find responses to your raised comments:

- Rewiever #1:
  - Regarding Length of hospital stay which is higher for open surgeries compared to laparoscopic procedures, we hypothesize differences are due to the well-known benefits of laparoscopy.
  - We confirm that costs of adhesions are probably underestimated since indirect costs are not included. Further studies would be interesting to produce.
  - References about benefits of anti-adhesion barriers after laparoscopic myomectomy have been well added in our manuscript which confirms interest of anti-adhesion barriers in all surgical approaches.
- Reviewer #2: No comment due to absence of question.
- Reviewer #3: No comment due to absence of question.

Furthermore, no funding was received from Baxter for any author of this paper. Please not that Dr Payen and Dr Lemaire were Baxter employees when the article was written, but they received no additional funding for this work apart of their monthly salary. Pr Fernandez and Dr Capmas did not receive any financial support from Baxter for this work.

Hoping that revised version will allow a future publication in “Plos one” review.

Yours faithfully.
